# Treatment of Pruritus Pudendi

**Published:** 1886-11

**Authors:** E. S. McKee

**Affiliations:** Cincinnati


### Clinical Reports.

### *TREATMENT OF PRURITUS PUDENDI.*

By E. S. McKEE, M. D., Cincinnati.

In a clinical lecture, in the Gynæcological Clinic of the Medical College of Ohio, the author discussed the treatment as follows, and gave the remedies here presented:

First we should ascertain the cause of the disease to treat it intelligently, we should treat the constitutional trouble, where we are apt to find the origin of the disease; next, we should treat the morbid phenomena, the pruritus. Remove the cause and the pruritus will disappear of itself. The parts should be washed twice a day with castile soap and water; the diet should be vegetable, and regular action of the bowels maintained; as a general rule stimulants should be disallowed. In this vexatious trouble, for we can hardly call it a disease, you will need all the remedies you can find. The following are the best:

| | | | | | |
|---|---|---|---|---|---|
| 4 | per | cent. | solution | of | Boracic Acid. |
| 2-10 | " | " | " | " | Carbolic Acid. |
| 2-5 | " | " | " | " | Argenti Nitratis. |
| 0-5 | " | " | " | " | Bichloride of Mercury. |
| 25-50 | " | " | " | " | Sulphurous Acid. |
| 6 | " | " | " | " | Sodii Biborat. |

Ointments of tar, boracic acid, camphor or iodoform, mixtures of camphor and chloral, infusions of tobacco, 20 per cent. solution of chloroform in almond oil.

Treatment with the bichloride should be preceded by a removal of the mucous with warm water, and then dry with a soft linen; pass a sponge moistened with the solution rapidly over the affected part. This leaves a smarting, burning sensation, which is alleviated by a few minutes washing with cold water; subsequent applications become less and less painful.

M. Dubois recommends, in the rebellious cases, that the entire surface of the vulva be cauterized with a solid stick of the nitrate of silver. The great objection to this is that it is extremely painful, and the alleviation produced by it is almost always temporary.

R. Meigs recommends:

| | | |
|---|---|---|
| ℞ | Borax, - - - - - | ʒ ii. |
| | Morph. Sulph., - - - | gr. iv. ss. |
| | Aquæ Rosæ Dest., - - - | ℥ viii. M. |

Sig.:—Apply three times a day to the affected part with a sponge or soft piece of linen; take care to wash well the parts beforehand with soap and warm water, and dry them well afterward. A compress dipped in the oil of sweet almonds and laid in the commisure of the vagina is recommended.

When the trouble is general, temporary relief may be obtained by placing the woman in a prolonged soda bath, and subsequently rubbing the entire surface with vaseline.

Pruritus which has extended upon the distended abdominal walls is well treated with:

| | | |
|---|---|---|
| ℞ | Lin. Saponis Comp., - - - | ℥ v. |
| | Chloroformi, - - - - | ℥ i. |

Sig.:—Apply locally.

If the itching comes from an ulcerated cervix, or more properly from the irritating discharge proceeding from it, apply nitrate of silver and introduce tampons of tanno-glycerine.

Pruritus from breeding pediculi is well treated by mild mercurial ointments; stavesacre answers well. A plasma formed of flowers of sulphur and water, saline purgatives as Pullna or Fredrickshall water, Vichy baths, or even bathing with cold or tepid water, constitutes the best palliatives; salines and colchicum may be indicated, also bromide of potassium, a weak solution of Goulard's lotion, or a lotion composed of:

| | | |
|---|---|---|
| ℞ | Liq. Morph. Hydrochlorate, - - | ℥ i. |
| | Acid Hydrocyanici, - - - | ʒ iss. |
| | Aquæ, - - - - - | ℥ vi. M. |

Sig.:—Use as a lotion.

